# Hydrothermally grown Cu doped NiMnO_3_ perovskite nanostructures suitable for optoelectronic, photoluminescent and electrochemical properties

**DOI:** 10.1038/s41598-024-52132-1

**Published:** 2024-03-28

**Authors:** Shilpi Upadhyay, Insaaf Assadullah, Radha Tomar

**Affiliations:** School of Studies in Chemistry, Jiwaji University, Gwalior, M.P 474011 India

**Keywords:** Chemistry, Materials science, Nanoscience and technology

## Abstract

Transition metal-based perovskites have emerged as highly promising and economically advantageous semiconductor materials due to their exceptional performance in optoelectronics, photovoltaic, photocatalysis, and photoluminescence. In this study, we employed a microwave-assisted hydrothermal process to produce a Cu-doped NiMnO_3_ nanocomposite electrode material. The appearance of a peak corresponding to the (110) plane with a 2θ value of 36.6° confirmed the growth of the rhombohedral NiMnO_3_ crystal structure. The presence of metal–oxygen bonds in NiMnO_3_ was confirmed through FTIR spectra. XPS validates the chemical composition, providing additional support for the results obtained from XRD and FT-IR analyses. FE-SEM affirmed the anisotropic growth of small sphere-like structures that agglomerated to form broccoli-like shapes. Cu doping modified the band gap, reducing it from 2.2 to 1.7 eV and enhancing its photoluminescent (PL) activity by introducing defects. The increase in PL intensity (visible light luminescent intensity) can be attributed to a concurrent rise in complex defects and the rate of recombination of electron–hole pairs. Finally, the electrochemical activity demonstrated the pseudo-capacitor behavior of the synthesized material, with capacitance values increasing as the copper (Cu) content in the parent lattice increased.

## Introduction

Humanity confronts an energy crisis stemming from finite fossil fuel reserves, population growth, and technological progress. Energy sources fall into two main categories: controlled man-made power plants and non-dispatchable renewables such as wind and solar energy. It’s becoming increasingly evident that sustaining the entire global population solely on renewable energy sources is not a feasible prospect^1^. Researchers worldwide have been developing materials, such as metal oxides, to address these challenges, and these materials have demonstrated effectiveness in this regard. With their remarkable power density and extended cycling lifespan, supercapacitors represent a distinctive and eco-friendly energy storage technology that has garnered substantial attention within the academic community^2–4^. Transition metal oxides are promising materials for a variety of uses, including urea oxidation, water oxidation, supercapacitors, hydrogen generation, and lithium-ion batteries^5^. However, due to their weak conductivity and scarce active sites, they only have a limited number of applications. Several strategies, like the addition of metal ions, the use of carbon-based materials, and the use of quantum dots, etc. have been used to try to solve these problems^6^ to boost the material’s significant properties, transition metal and rare earth metal-doped metal oxides in particular display exceptional activity^7^. Through doping and creating composites, many transition metals have been employed to improve the properties of parent metal oxides. This has significantly improved ionic conductivity, electrochemical performance, photoluminescent performance, and carrier transportability^8,9^. There are now numerous ways to make metal perovskite nanostructures, including, sol–gel methods, co-precipitation, hydrothermal, and microwave-assisted processes^10^. The hydrothermal method is one of the several techniques for creating available nanostructures, and it offers many benefits such as low operating temperatures, simple instrumentation, and ease of use. It enables materials to develop and nucleate uniformly^11^. NiMnO_3_ has been extensively explored in various disciplines due to its benefits, which include high chemical and physical activity, an abundance of resources, and non-toxicity. Thus, they have received much research across several disciplines. Particularly, in the field of supercapacitors^12^, electrocatalysts^13^, water splitting^14^, and photoluminescent characteristics^15,16^ by numerous researchers. For example, Mustafa Al Samarai et al. synthesized a graphene-supported Ni_3_MnO_4_ catalyst by a reverse micellar method for electrocatalytic OER^17^. Zhang et al. developed nickel manganese composite oxide nanomesh using the hydrothermal process, then performed electrochemical water oxidation and high-temperature calcination for application in supercapacitors^18^. Ji et al. fabricated carbon-based nickel and manganese oxide nanoparticles via the electrospinning-calcination method for electrochemical water oxidation^19^. Kakvand et al. studied the synthesis of NMO/C through the co-precipitation method and reported Cs of 285 Fg^−1^ at 1 Ag^−1^ and 93.5% cycle stability after 1000 cycles^20^. Ge et al. Giri et al. developed an NMO/nitrogen-doped graphene nanocomposite, which provided Cs of 523.5 Fg^−1^ and 82.31% cycle stability for 1000 cycles at a current density of 1 Ag^−1^^21^. Sanchez’s group observed NiMnO_3_-rGO nanocomposites to be excellent electrode materials with a high capacity of 91 mAh g^−1^ at a 5 mV s^−1^ scan rate^22^. A study by Faraji’s group examines metal oxide/hydroxide composite electrodes as high power supercapacitors with microwave assistance^23^. Kim’s team concentrated on using mixed oxides that used MnO_2_ as supercapacitor electrode material^24^. Therefore, electrode materials based on Ni–Mn metals are considered potential candidates and the key challenges lie in enhancing and improving their properties while keeping costs affordable In our current study, we successfully report the synthesis of NiMnO_3_ and modified Cu-doped NiMnO_3_ nanostructures under subcritical conditions using a hydrothermal technique. This synthesis process was completed in a remarkably short reaction time of 4 h, compared to the previously employed duration of more than 4 h. We have managed to avoid the use of toxic and costly chemicals in this fabrication process. Furthermore, the introduction of copper as a doping agent has not only altered the electrical characteristics of the material but also enhanced its physio-chemical properties, including optical, luminescent, and charge storage properties, when compared to pristine.

## Experimental details

### The preparation of NiMnO_3_

**Figure 1:**
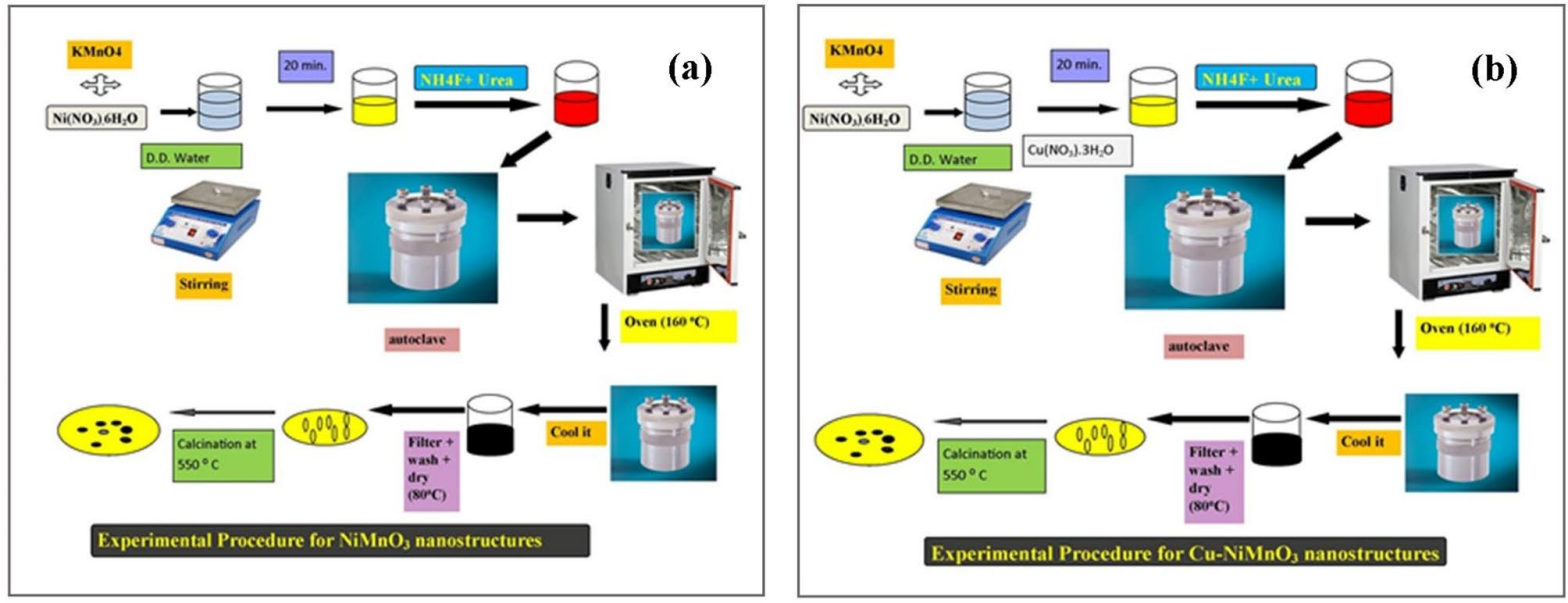
(**a**) Represents the schematic route for the synthesis NiMnO_3_ and (**b**) Cu-doped NiMnO_3_.

The analytical grade of each chemical reagent allowed for use without further purification. The hydrothermal approach was used to create the NiMnO_3_ nanostructures shown in Fig. 1a. Nickel nitrate (Ni(NO_3_)_2_·6H_2_O) and potassium permanganate (KMnO_4_) respectively, were chosen as the starting ingredients for nickel and manganese, respectively. The following is a typical synthetic process for creating nanostructures. 0.1 M Ni(NO_3_)_2_·6H_2_O and 0.1 M KMnO_4_ were dissolved in 30 mL of double-distilled water while continuously stirring for 20 min. Next, 0.6 M NH_4_F and 1.2 M urea were added, and stirring continued for 15 min. The solution was then treated at 160 °C for 4 h before being moved to an autoclave lined with Teflon created specifically for microwave heating. After allowing the sample to cool to ambient temperature, it was filtered and repeatedly washed with ethanol and distilled water. The collected dark green color sample was annealed at 550 °C for 3 h after being dried at 80 °C overnight. The dark black color powder, which mimics the previously described papers, was finally acquired^25^.

### Preparation of Cu-NiMnO_3_

For the fabrication of Cu-doped NiMnO_3,_ as shown in Fig. 1b, the nickel nitrate and potassium permanganate solutions were mixed. Then Cu (NO_3_)_2_·3H_2_O with different weight percentages such as 5%, 7%, and 10% was added to the former reaction solution followed by adding 0.6 M NH_4_F and 1.2 M urea. The whole solution was stirred for 15 min and then transferred to Teflon coated autoclave and put in a hot air oven for just 4 h at 160 °C. Cool the autoclave naturally and filter the fabricated sample which was washed several times with condensed water and ethanol. Collect the nanostructures for drying and were annealed at 550 °C for about 3 h.

### Characterization

**Figure 2:**
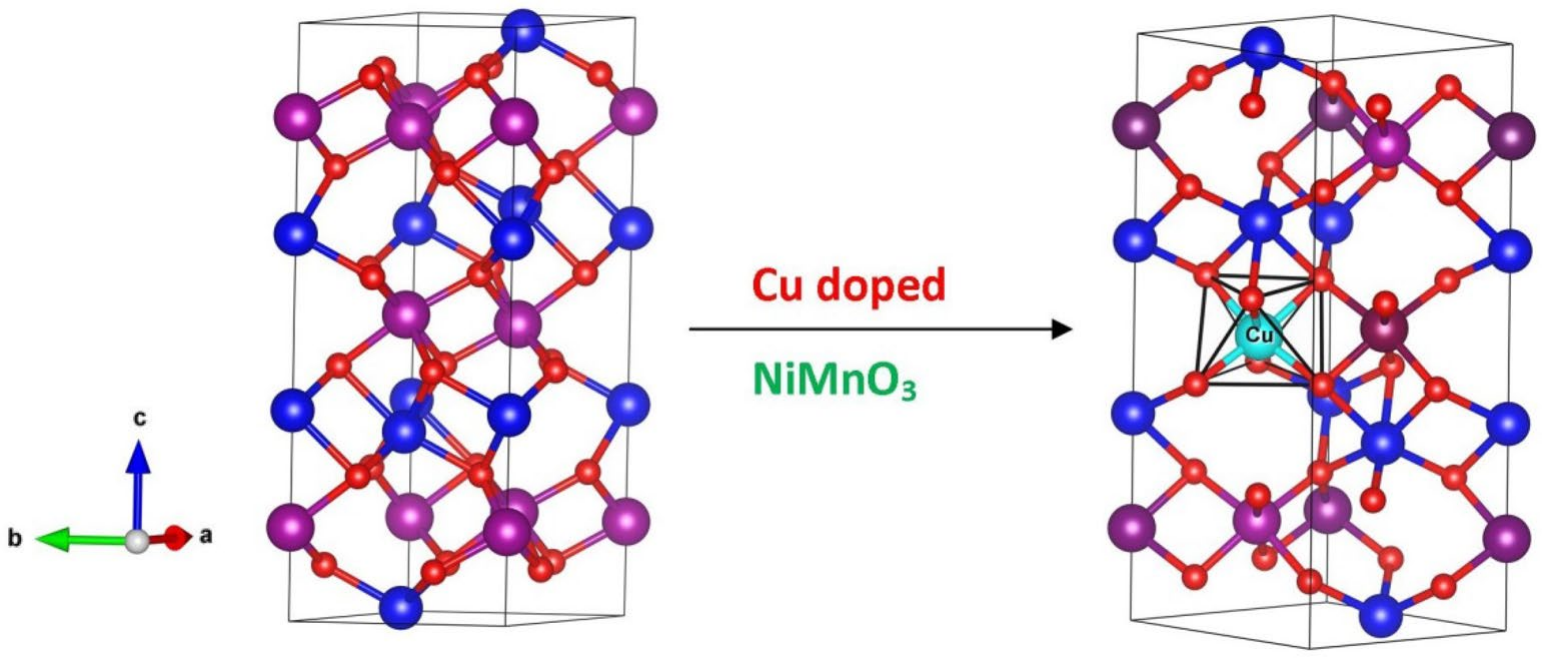
Crystal structure of fabricated perovskite nanostructures.

The amalgamated particles were subjected to physical, morphological, compositional, and optical studies. The structural properties of Cu-doped NiMnO_3_ were determined using the X-ray diffraction (XRD, Rikagu Mini-flex 600 with Cu-Kα radiation) technique. Morphological properties were examined using a FESEM (Philips, Model-Quanta 200 FEG). X-ray photoelectron spectroscopy (XPS) analysis was performed utilizing the Thermo ESCALAB 250 XPS system with Al-Kα radiation. The optical absorbance of QDs was explored using a UV–Vis spectrophotometer (UV 2450 Shimadzu) and for photoluminescence, a spectrofluorophotometer (RF 6000, Shimadzu) and for CV, a PG stat 204 was employed. The crystal structure of NiMnO_3_ and Cu doped NiMnO_3_ perovskite nanostructures are displayed in the Fig. 2.

## Results and discussion

**Figure 3:**
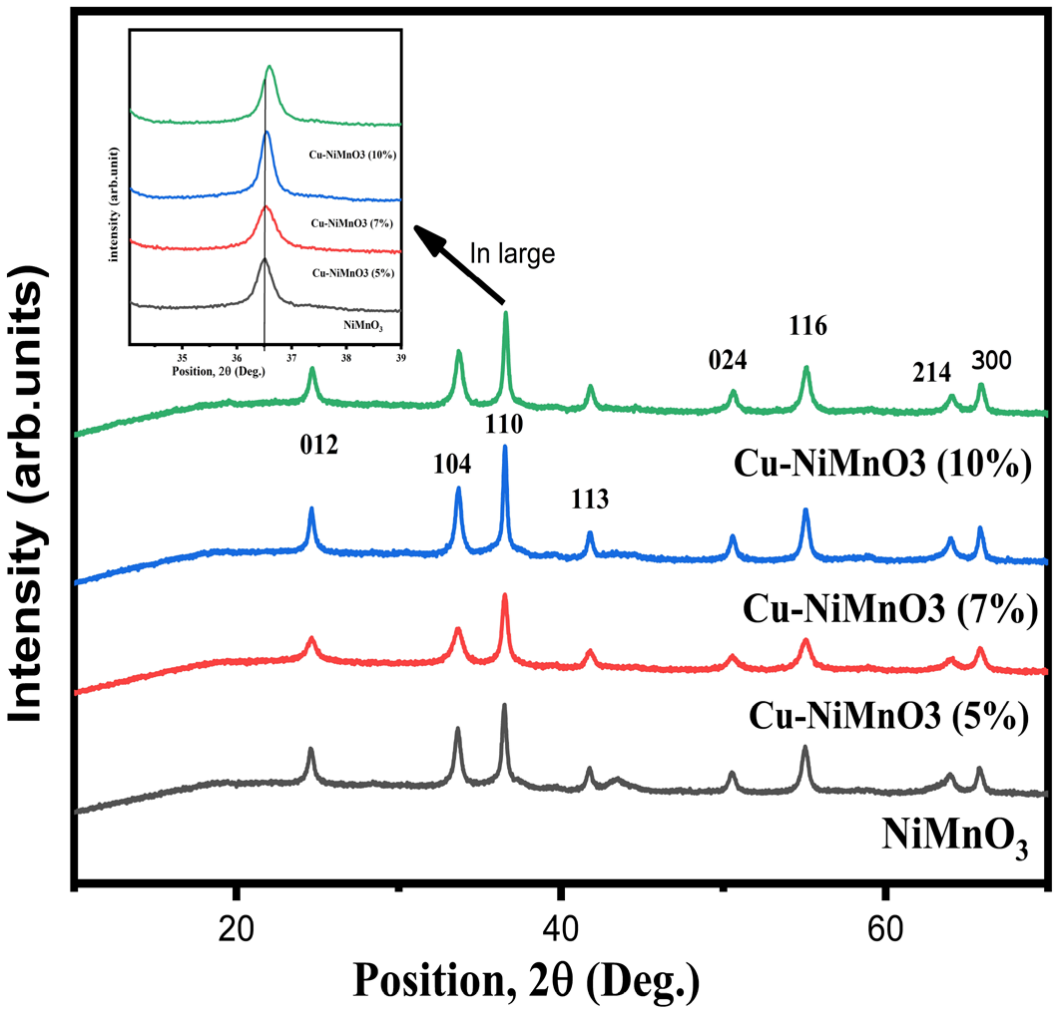
XRD patterns of the Pristine and Cu-doped NiMnO_3_ nanostructures.

The crystal structure and function groups of the obtained samples NiMnO_3_ and modified Cu-doped NiMnO_3_ are presented in Fig. 3. From the patterns it is clear that the strong crests occurred at 24.7, 33.7, 36.6, 41.8, 50.6, 64, and 65.9 degrees, and consequently the corresponding crystal planes are (012), (104), 110), (113), (024), (116), (214) and (300) of NiMnO_3_ which resemble well with the previously reported data respectively. These Miller indices look into the R3 spatial symmetry of the rhombohedral crystal phase (JCPDS No. 75-2089)^26^. The lattice constant of NiMnO_3_ material are [(a = 4.91), (b = 4.91), (c = 13.58)]^27^. The substitution of Cu metal for Mn in the perovskite lattice induces strain due to alterations in ionic radii. This strain can manifest as either compressive or tensile, discernible through variations in the 2θ value. An increase in the 2θ value is indicative of compressive strain, whereas a decrease suggests the presence of tensile strain. The diffraction peak’s very slight shift to higher 2 theta values than the NiMnO_3_ is visible from the XRD image. That occurred as a consequence of the sample’s compressive strain^28,29^. These diffraction peaks indicate that NiMnO_3_ has been effectively prepared by the microwave-assisted hydrothermal method. The sharpness of the major peaks’ maximums illustrates the high crystallinity of NiMnO_3_ nanoparticles. No, hydrated NiMnO_3_ peaks were discovered in the sample, demonstrating the high purity of NiMnO_3_ and its altered forms by doping with various weight percentages of Cu. The crystallite size was calculated by using the Scherer formula.

$${\text{D}} = 0.9 \times\uplambda /\upbeta \cos\uptheta$$

here D is the average size of the nanoparticles, n is the dimensionless shape factor (0.9), λ is the wavelength of incident X-ray (λ = 1.54 Å), β is the full width at half maximum (FWHM) of the diffraction peak and θ is the angle of diffraction^28,29^. As the concentration of Cu content increases, there is a lessening in the crystallite size leading to an increase in lattice strain because of compressive strain and an increase in the dislocation density^30^. The dislocation density, lattice strain, and crystallite size are displayed in Table 1.

**Table 1:** Shows the crystallite size, dislocation density (nm^−2^), and lattice strain.

Serial no.	Sample name	Crystallite size	Dislocation density	Lattice strain
1	NiMno_3_	29.422	1.3312361E−6	.00298
2	Cu-NiMno_3_ (5%)	27.689	1.7013232E−6	.00321
3	Cu-NiMno_3_ (7%)	22.962	3.5927881E−6	.00399
4	Cu-NiMno_3_ (10%)	14.046	2.5691493E−5	.00468

### FTIR analysis

**Figure 4:**
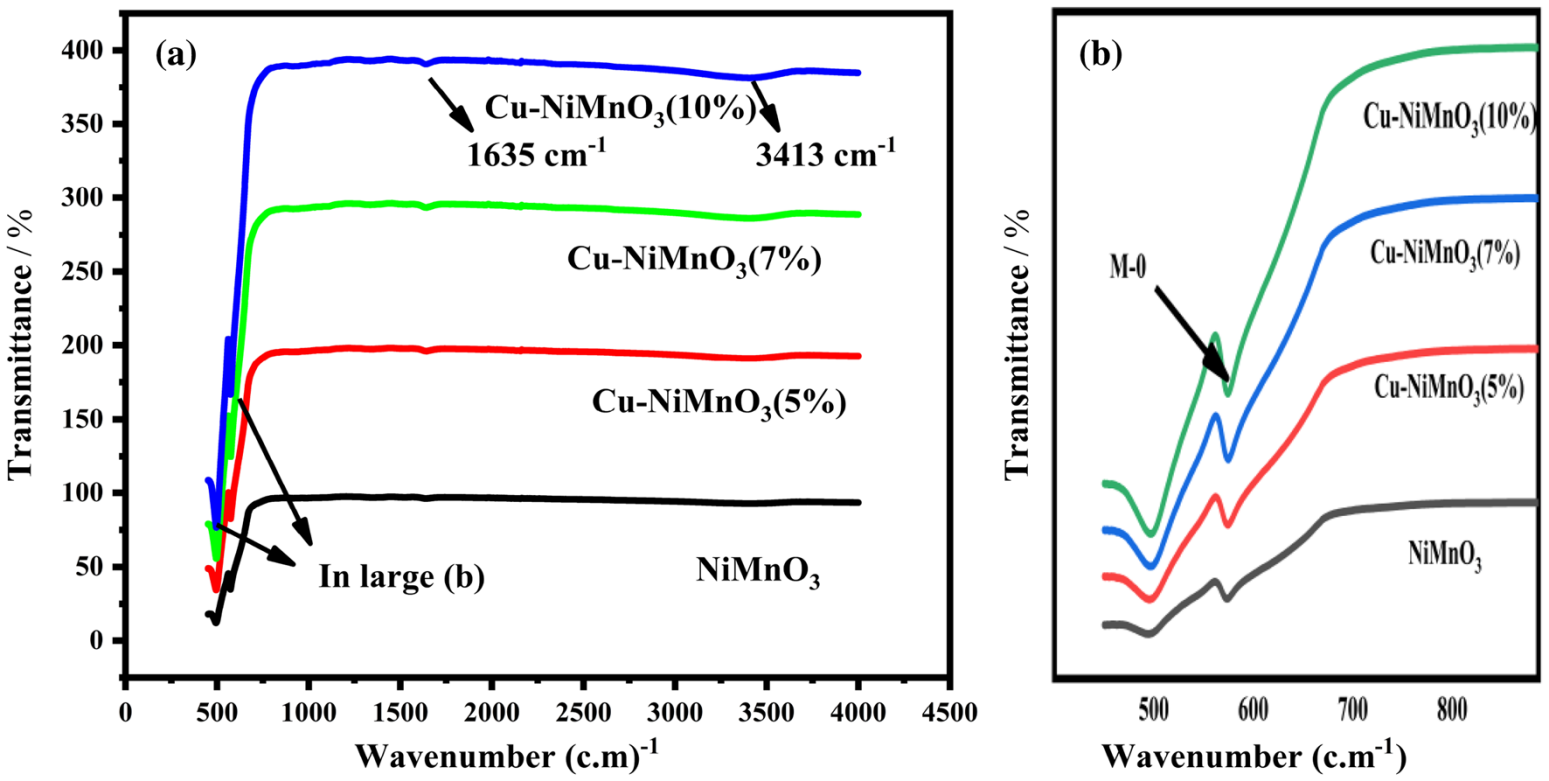
(**a**,**b**) FTIR spectra of NiMnO_3_ and Cu-doped NiMnO_3_ nanostructure.

FTIR spectroscopy of the created NiMnO_3_ nanoparticles was carried out at room temperature to examine the chemical composition and to identify the many associated distinctive functional groups. The FTIR spectra of pure NiMnO_3_ and Cu-doped NiMnO_3_ at several weight percentages (5%, 7%, and 10%) have been exhibited in Fig. 4a,b. From the graphs, it is revealed that the bands present below 1000 cm^−1^ displayed in the figure at 497 cm^−1^ and 585 cm^−1^ corresponded to NiMnO_3_ which indicates a metal–oxygen bond while the band present at 1635 cm^−1^ and 3413 cm^−1^ demonstrate the H–O–H vibrations of water which is present in NiMnO_3_ and Cu doped NiMnO_3_, the introduction of Cu atom can leads to change in the intensity of these peaks, which is clearly demonstrated in Fig. 3b, no other peaks of impurity is present thus indicate a synthesis of NiMnO3 and the incorporation of Cu was successfully done^31,32^.

### Morphological analysis

**Figure 5:**
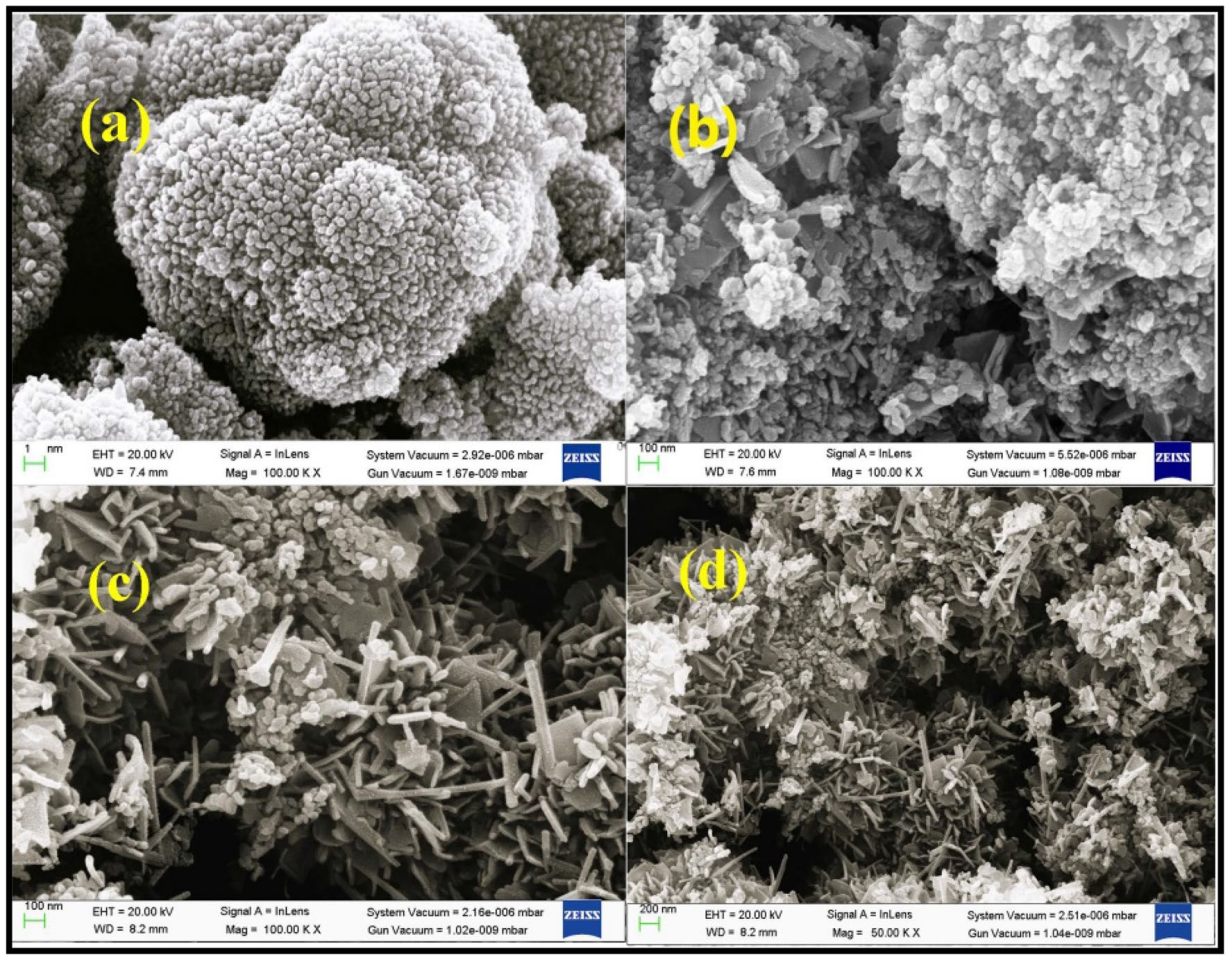
Micrographs of the Pristine and Cu-doped NiMnO_3_ nanostructures.

The morphology and particle size of the sample were studied by Field emission scanning electron microscopy. Figure 5a–d represents the FESEM micrographs of NiMnO_3_ (a), Cu-NiMnO_3_ (b), (c), (d) 5%, 7% and 10% respectively. NiMnO_3_ nanoparticles have irregular Nano sphere-like structures the images show agglomeration of nanoparticle-over one another and resulting in the formation of broccoli-like structures. This is due to the anisotropic growth of the nanostructures.While Cu nanoparticles have tube-like structures. It is clear from the FE-SEM images that the Cu nanoparticles are incorporated on the surface of the parent material, thus increasing the surface area of the martial which is responsible for stimulating the properties of the modified nanostructures.

**Figure 6:**
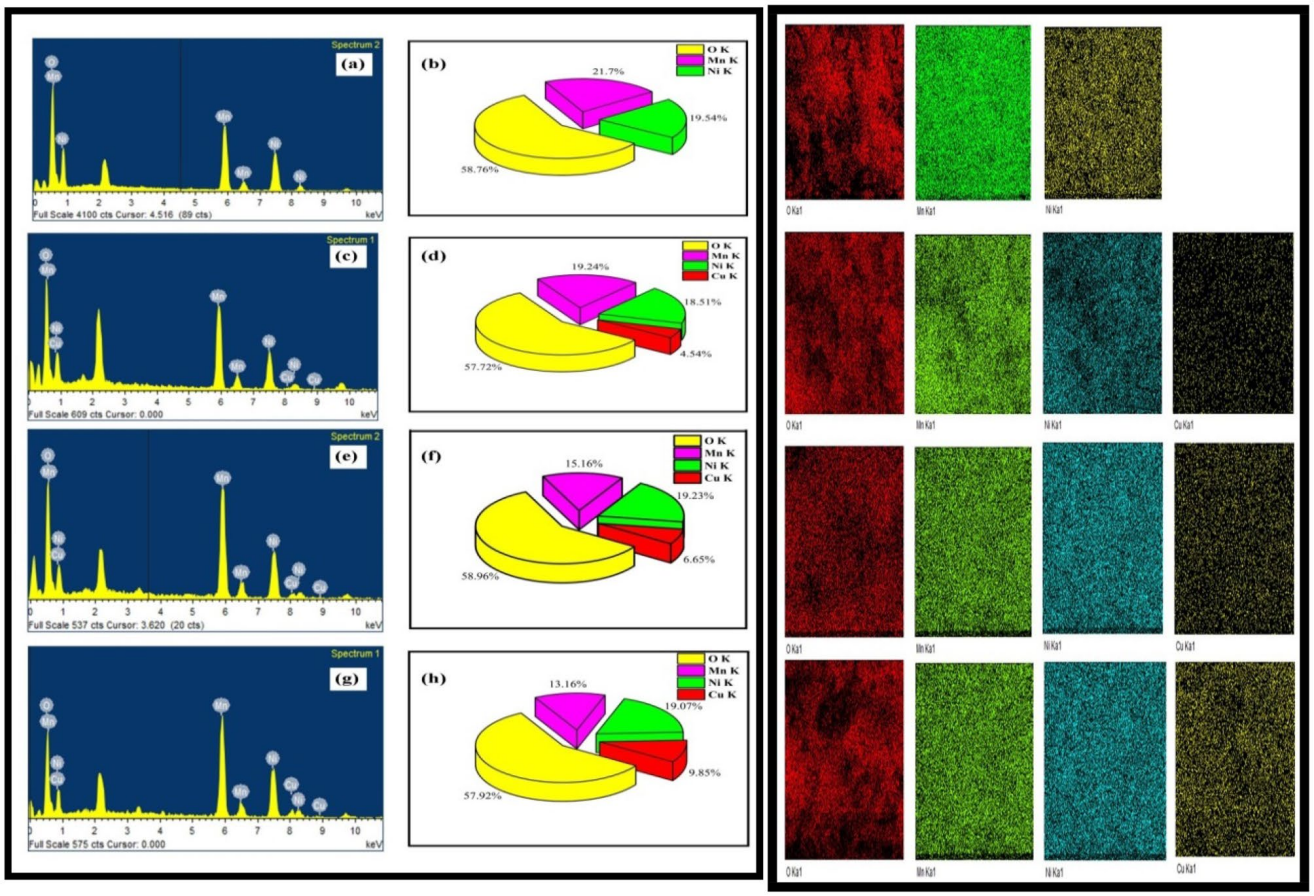
Left shows EDS, elemental composition, and elemental mapping of NiMnO_3_ (**a**,**b**), Cu-NiMnO_3_ (5%) (**c**,**d**), and Cu-NiMnO_3_ (7%) (**e**,**f**), Cu-NiMnO_3_ (10%) (**g**,**h**), elemental mapping (right) of parent and modified NiMnO_3_ perovskite nanostructures.

To determine the elemental composition of the fabricated samples, Energy dispersive spectrometry (EDS) was employed. The EDS arrangements and elemental composition of NiMnO_3_ and Cu-doped NiMnO_3_ at different weight percentages are displayed in Fig. 6a–h (left) respectively. The main elements in the samples were Ni, Mn, O, and Cu. No other elements were detected, thus, displaying the high transparency of the sample. Weight percentages of Cu gradually increase with the decreasing weight percentages of Mn which clearly show that Cu replaces Mn ions. Inorder to further ascertain in the distribution of components in the parent and modified perovskite nanostructures elemental mapping was employed displayed in Fig. 6 (right). The study revealed that all the components are distributed uniformly throughout the sample.

### X-ray photoelectron spectroscopy (XPS) analysis

**Figure 7:**
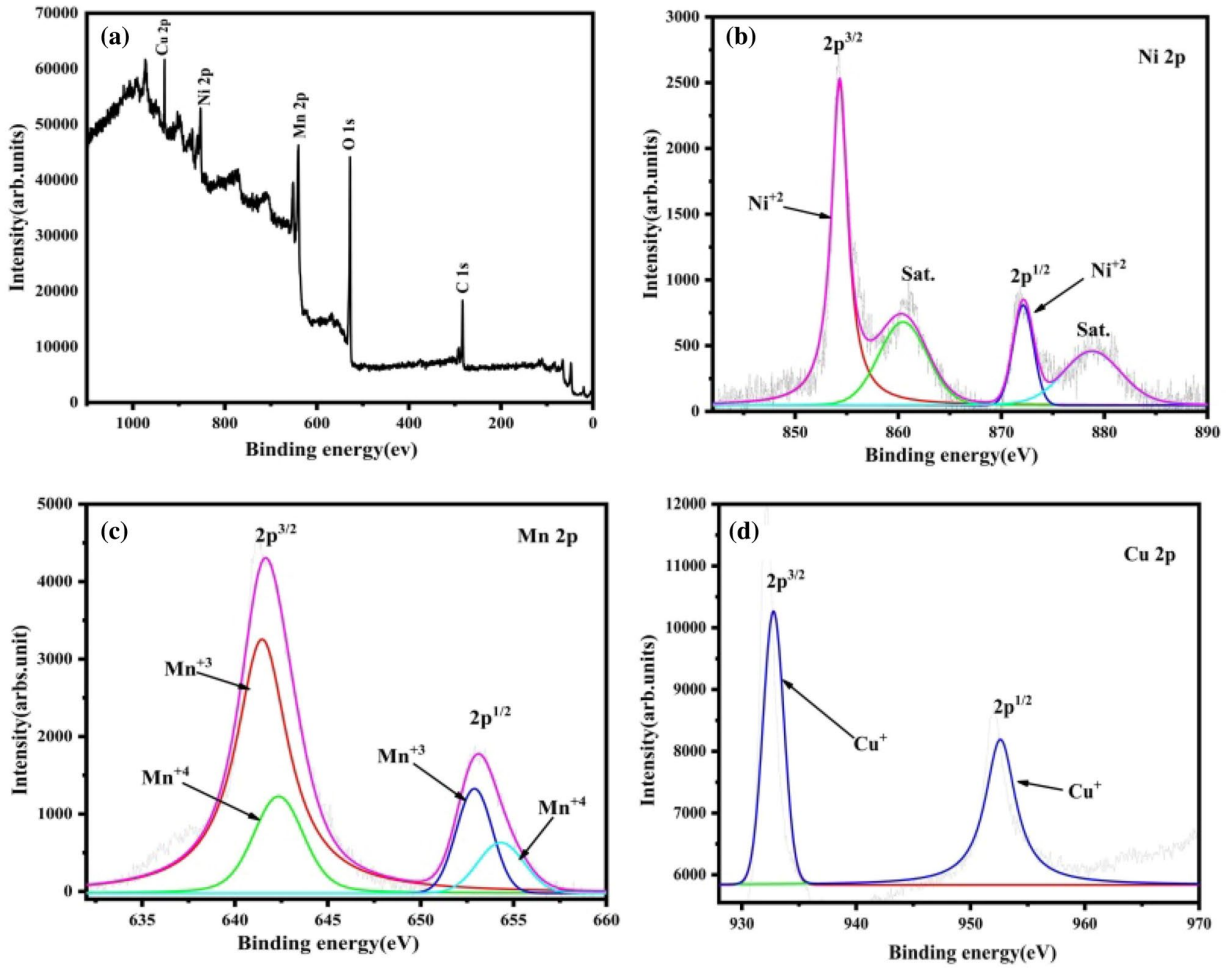
XPS spectra of 10% Cu-doped NiMnO_3_: (**a**) survey spectrum, (**b**) Ni 2*p*, (**c**) Mn 2*p*, and (**d**) Cu 2*p*.

The chemical composition of NiMnO_3_ materials doped with 10% Cu is illustrated in Fig. 7a–d. The survey spectra (Fig. 7a) reveal prominent peaks corresponding to C 1*s*, O 1*s*, Mn 2*p*, Cu 2*p*, and Ni 2*p*, with no additional discernible peaks; this suggests the successful incorporation of Cu ions into the NiMnO_3_ electrode materials. In Fig. 7b, the Ni 2*p* peak is depicted, with distinct 2*p*^3/2^ and 2*p*^1/2^ peaks observed at 854.20 and 871.68 eV, respectively. Through a fitting method, the Ni^2+^ oxidation state was determined to be at 854.31 and 871.73 eV, with no presence of other oxidation states. Applying the same method to the Mn 2*p* peak in Fig. 7c, the 2*p*^3/2^ and 2*p*^1/2^ peaks were observed at 641.71 and 653.26 eV, respectively. Mn^3+^ was identified at 641.45 and 652.89 eV, and Mn^4+^ was identified at 642.34 and 654.31 eV. Regarding the Cu 2*p* peak in Fig. 7d, the 2*p*^3/2^ and 2*p*^1/2^ peaks were observed at 932.18 and 952.085 eV, respectively. Cu^+^ was identified with binding energies of 932.76 and 952.61 eV^33–36^. The X-ray photoelectron spectroscopy (XPS) findings affirm the successful synthesis of the Cu-doped NiMnO_3_ electrode material, consistent with both X-ray diffraction (XRD) and Fourier-transform infrared spectroscopy (FT-IR) results.

### Optical analysis

**Figure 8:**
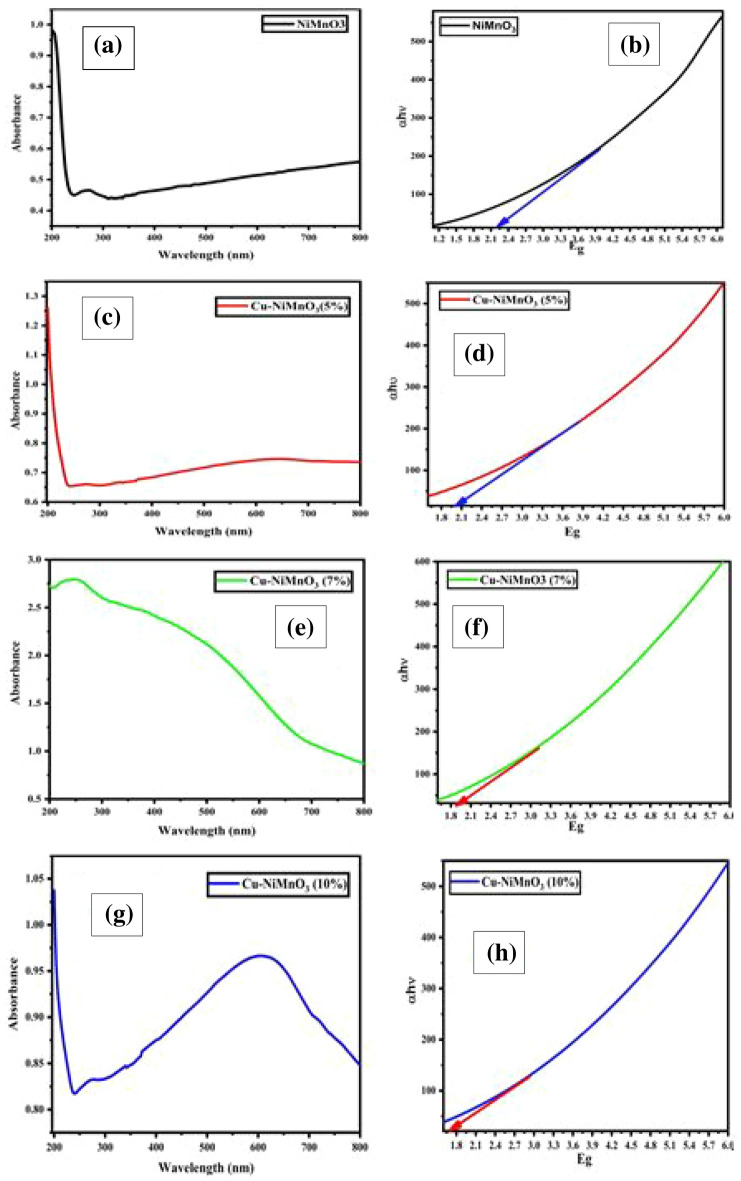
(**a**,**c**,**e**,**g**) Show optical absorbance and (**b**,**d**,**f**,**g**) Band-gap analyses using the relevant Tauc plots.

Optical absorption was identified using a UV–visible spectrophotometer in the wavelength range of 200–800 nm. The samples exhibit good UV–visible absorption in the electromagnetic spectrum displayed in Fig. 8a,c,e,g. The samples’ direct band-gap values were calculated from the absorbance spectra using the wood and Tauc plots depicted in Fig. 8b,d,f,h, and the band-gap values of the fabricated are around 2.2, 2.0, 1.9, and 1.7 eV respectively which quite resemble with the previously reported data^37^. The presence of dopant atoms (Cu) introduces additional energy levels within the bandgap. These energy levels create new electronic states making it easier for electrons to transition from the valence band to the conduction band hence the ease of electrons transition reduces the band gap value^38,39^,which can modify the conductivity and optical characteristics of a material. The pure NiMnO_3_ sample shows absorption in the UV range. The absorption bands near 200 nm are associated with ligand metal charge transfer (LMCT) of O^2−^ → Mn^4+^ in the octahedral environment^40^. As the concentration of doping increases, it shows significant enhancement in the visible region. The Cu doping may introduce new energy levels in the NiMnO_3_ band structure, which can cause the emergence of additional peaks in the UV–visible spectrum. The additional bands at 550 and 600 nm corresponded to the ligand metal charge transfer of O^2−^ → Cu^2+^ as observed for CuO^41^.

### Photoluminescence

**Figure 9:**
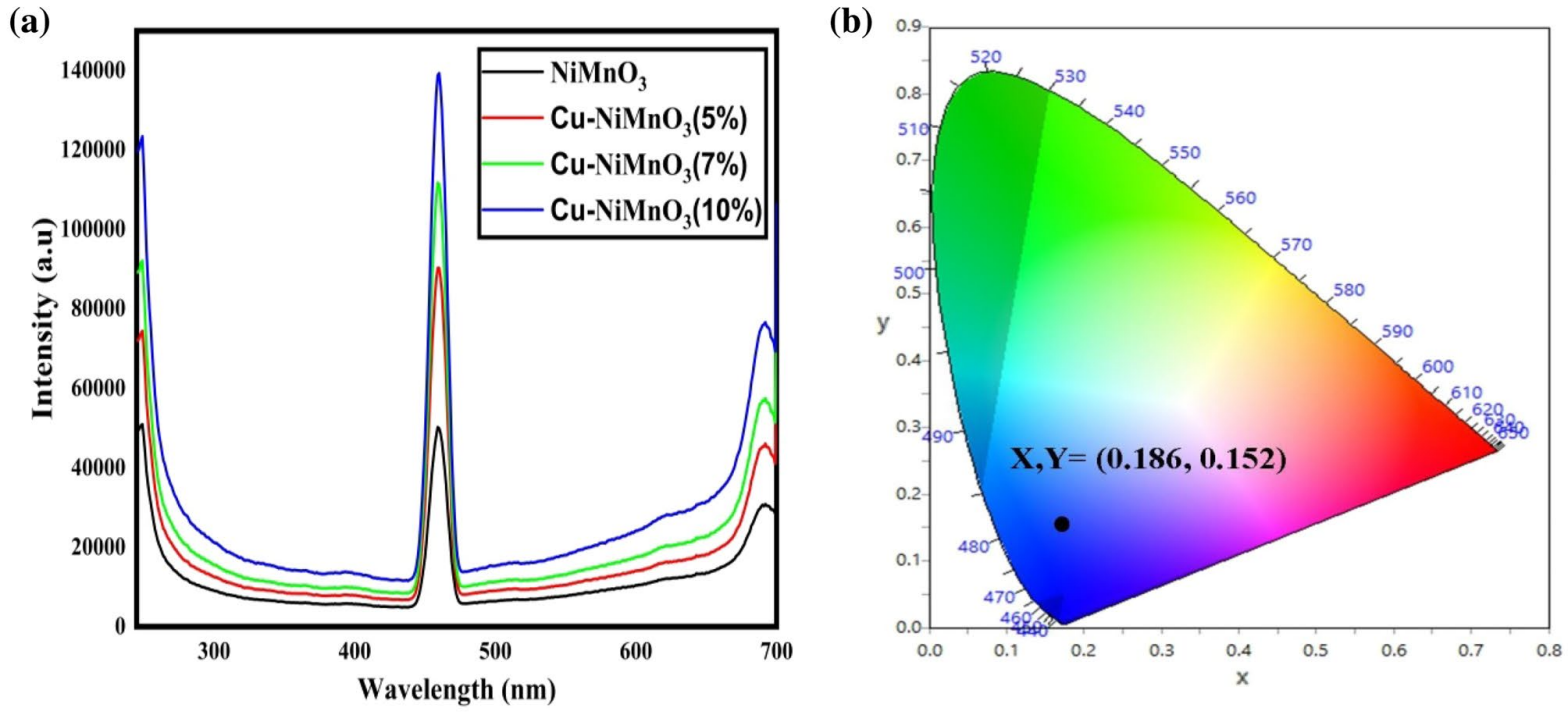
(**a**) PL spectra (**b**) CIE diagram of Cu doped NiMnO_3_.

Photoluminescence (PL) spectroscopy is a extensively utilized technique to examine the material features of perovskites, including their bandgap, electronic defects, phase distribution, local disorder, and dynamic phenomena^42^. Figure 9a displays the samples’ PL spectra viewed at room temperature. When the samples were excited by photons of wavelength 230 nm, the spectra had three peaks. The “near-band-edge emission” (NBE) refers to the emission of light from a material that is generated by the recombination of excited electrons with holes near the band edge of the material is seen at 249 nm. It is evident from the PL emission that the presence of dopant atoms causes a tiny blue shift in the Pl emission, which is caused by the substitution of Cu atoms for Mn atoms in the host lattice visible at 461 nm; because of this replacement lattice contraction occurs in the parent material^43,44^. Figure 9b shows the CIE diagram of the nanomaterial which shows the color coordinate at (X = 0.186, Y = 0.152). Hence such type of nanomaterials may be used in the application of blue emission LED’s like mobile phone, computer, flat TV. At 693 nm a broad peak appeared and this peak may occur due to the generation of intermediate energy level that causes the reduction in the energy gap of the material which results the emission at longer wavelengths. This can lead to the appearance of a broad band or peak at the end of the spectrum.

### Electrochemical properties

**Figure 10:**
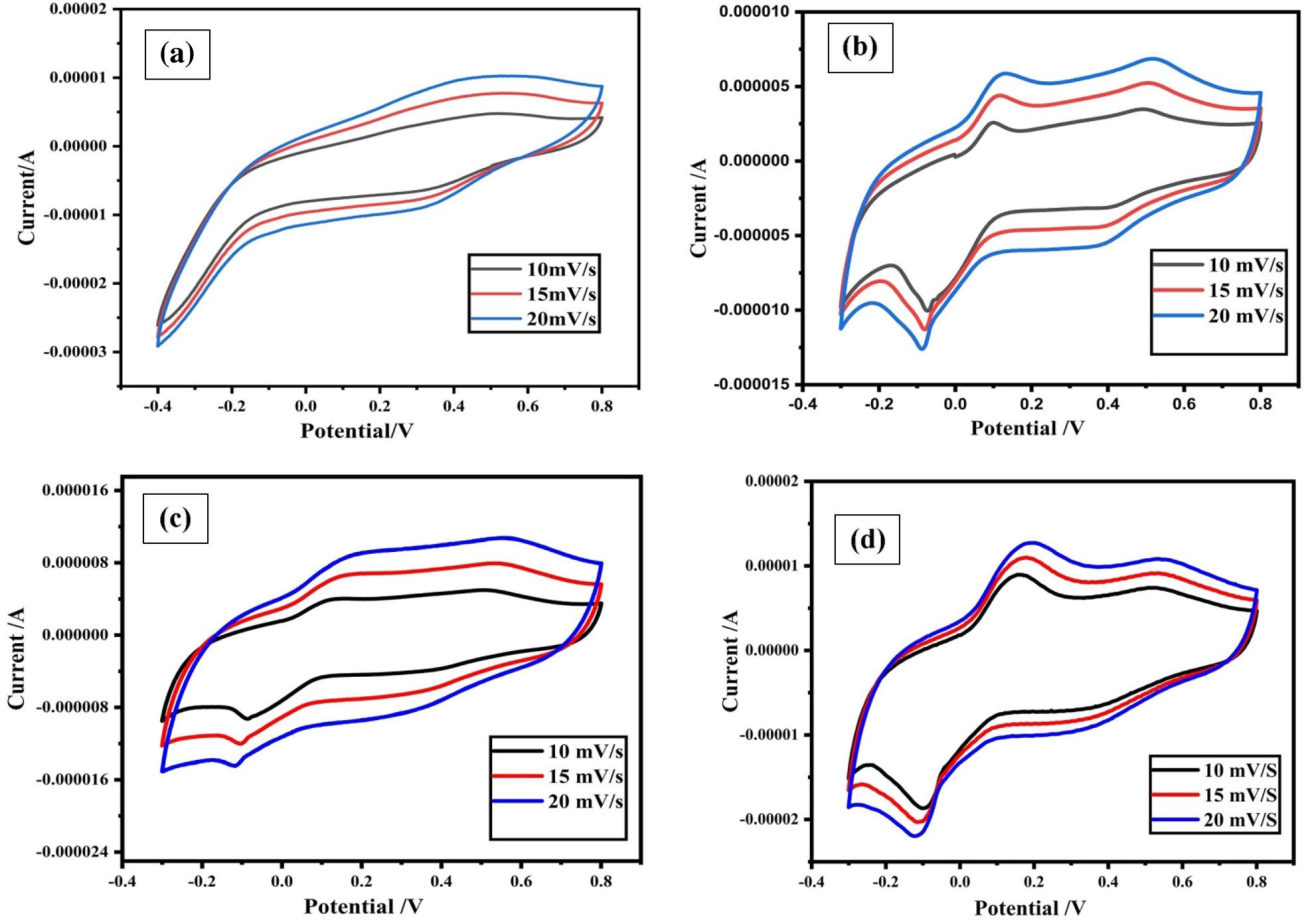
Show CV graphs of (**a**) NiMnO_3_ and (**b**–**d**) are Cu doped (5, 7 and 10%) respectively.

The electrochemical performance of NiMnO_3_ and Cu-doped NiMnO_3_ was determined at room temperature across a potential window of − 0.30 V to 0.79 V in 0.5 mol/L H_2_SO_4_ electrolyte (Fig. 10a,b,c,d). The three electrodes used in the CV setup are GCE (glassy carbon electrode) as the working electrode, Pt wires as the counter electrode, and Ag/AgCl electrode as the reference electrode. The voltammogram’s have a distinct oxidation and reduction peaks. The redox reaction of M–O/M–O–OH (M = Mn, Cu) is the primary cause of two couples of redox peaks. The redox peak of Mn^3+^/Mn^4+^ and Cu^+^/Cu^2+^ are separated because MnO_2_ and CuO have distinct redox voltages. Because of this, it is clear from the CV shapes’ that the samples NiMnO_3_ and Cu-doped NiMnO_3_ are made of pseudo-capacitive material^45,46^. Consequently, these capacitors can be utilized to create supercapacitors because they have the highest charge storage capacity. The formula can be used to determine the specific capacitance of any material.

1 $$Cs = \frac{A}{{\left[ {mk\left( {V_{2} - V_{1} } \right)} \right]}}$$

where A is the Voltammogram’s surface area, m is the mass dropped onto the working electrode, k is the scan speed, and (V_2_–V_1_) is the potential window^47^. According to Eq. (1) the evaluated specific capacitance of NiMnO_3_ and Cu doped NiMnO_3_ at (5, 7, and 10%) are 257.1 F g^−1^, 302.30 F g^−1^, 394.25 F g^−1^, and 659.50 F g^−1^ respectively displayed in Fig. 9a. It was found that 10% Cu-doped NiMnO_3_ had a high specific capacitance. An increase in the integrable area of the Voltammogram, which reflects the larger storage capacity, is shown as the concentration of dopant rises. As the scan rate rises, the material’s storage capacity also decreases because the interaction between the electrode and electrolyte must not be given sufficient time.

**Figure 11:**
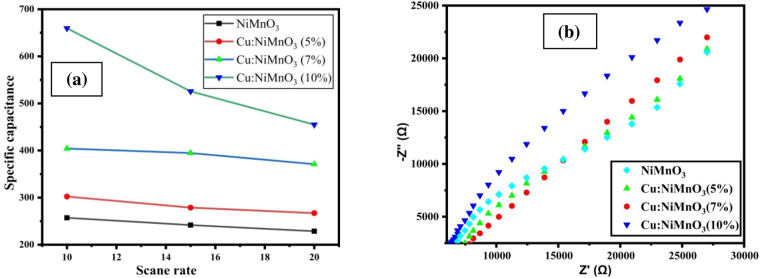
(**a**) Shows the specific capacitance at the different scan rate and (**b**) shows the Nyquist curve.

The Nyquist curve of NiMnO_3_ and Cu-doped NiMnO_3_ is displayed in Fig. 11b. All of the samples display practically a straight line in the low-frequency region, which is generated by the frequency dependence of ion transportation from the electrolyte on the surface known as the Warburg resistance. The 10% Cu doped NiMnO_3_ perovskite nanostructures revealed lowered resistance values, thus showed the higher capacitance values from the other counterparts^48^. Therefore, this material showed incredible electrochemical properties as the specific capacitance of this material is moderately higher than the other fabricated nanostructures.

## Conclusion

In this study, we present the synthesis of modified Cu-doped NiMnO_3_ perovskite nanostructures through a hydrothermal route, conducted under subcritical conditions for a duration of 4 h. Our X-ray diffraction analysis revealed a significant enhancement in crystallinity, characterized by a rhombohedral structure with an R3 spatial group. Field emission Scanning electron microscopy (FE-SEM) imaging showed that the pristine nanostructures exhibited a spherical morphology, while the Cu-doped nanostructures demonstrated a tubular structure. The introduction of Cu dopants resulted in a reduction of the bandgap in the modified nanostructures, thereby improving their optoelectronic properties. These enhanced nanomaterials hold great promise for various applications, displaying not only improved crystallinity but also remarkable luminescent and electrochemical performance, as confirmed through cyclic voltammetry investigations. Specifically, these materials are well-suited for blue light-emitting diodes (LEDs), meeting the International Commission on Illumination (CIE) color coordinates of (X, Y = 0.186, 0.152) for blue emission, making them suitable for incorporation into mobile phones, flat-screen TVs, and blue LEDs.

## Data Availability

The data that supports the outcomes of this study are available from the corresponding author upon reasonable request.
